# Vesiculopolins, a New Class of Anti-Vesiculoviral Compounds, Inhibit Transcription Initiation of Vesiculoviruses

**DOI:** 10.3390/v11090856

**Published:** 2019-09-14

**Authors:** Minako Ogino, Yuriy Fedorov, Drew J. Adams, Kazuma Okada, Naoto Ito, Makoto Sugiyama, Tomoaki Ogino

**Affiliations:** 1 Department of Molecular Biology and Microbiology, Case Western Reserve University School of Medicine, Cleveland, OH 44106, USA; 2 Small Molecule Drug Development Core, Case Western Reserve University School of Medicine, Cleveland, OH 44106, USA; 3 Department of Genetics and Genome Sciences, Case Western Reserve University School of Medicine, Cleveland, OH 44106, USA; 4 Laboratory of Zoonotic Diseases, Faculty of Applied Biological Sciences, Gifu University, 1-1 Yanagido, Gifu 501-1193, Japan; 5 Gifu Center for Highly Advanced Integration of Nanosciences and Life Sciences (G-CHAIN), Gifu University, 1-1 Yanagido, Gifu 501-1193, Japan; 6 Department of Inflammation and Immunity, Lerner Research Institute, Cleveland Clinic, Cleveland, OH 44195, USA

**Keywords:** vesicular stomatitis virus, Chandipura virus, vesiculoviruses, L protein, RNA-dependent RNA polymerase, transcription, small molecule inhibitor, oncolytic viruses, vaccine vectors

## Abstract

Vesicular stomatitis virus (VSV) represents a promising platform for developing oncolytic viruses, as well as vaccines against significant human pathogens. To safely control VSV infection in humans, small-molecule drugs that selectively inhibit VSV infection may be needed. Here, using a cell-based high-throughput screening assay followed by an in vitro transcription assay, compounds with a 7-hydroxy-6-methyl-3,4-dihydroquinolin-2(1H)-one structure and an aromatic group at position 4 (named vesiculopolins, VPIs) were identified as VSV RNA polymerase inhibitors. The most effective compound, VPI A, inhibited VSV-induced cytopathic effects and in vitro mRNA synthesis with micromolar to submicromolar 50% inhibitory concentrations. VPI A was found to inhibit terminal de novo initiation rather than elongation for leader RNA synthesis, but not mRNA capping, with the VSV L protein, suggesting that VPI A is targeted to the polymerase domain in the L protein. VPI A inhibited transcription of Chandipura virus, but not of human parainfluenza virus 3, suggesting that it specifically acts on vesiculoviral L proteins. These results suggest that VPIs may serve not only as molecular probes to elucidate the mechanisms of transcription of vesiculoviruses, but also as lead compounds to develop antiviral drugs against vesiculoviruses and other related rhabdoviruses.

## 1. Introduction

Vesicular stomatitis virus (VSV, an animal vesiculovirus belonging to the *Rhabdoviridae* family) has served as a paradigm for studying the molecular mechanisms of transcription and replication by nonsegmented negative strand RNA viruses (e.g., rabies virus (RABV), measles virus, Ebola virus). VSV has now become a clinically important virus that has the potential to be developed as an oncolytic virus [1,2,3,4,5,6] as well as a promising vaccine vector against human pathogens, such as Ebola virus [7,8,9]. However, safety concerns remain regarding potential side effects (e.g., viraemia, arthritis, conjunctivitis, oral ulcers, dermatitis, vesicle lesions, and encephalitis) caused by possible replication of VSV in peripheral organs [8,10,11] and potentially the brain [12,13,14,15]. Although genetic engineering of recombinant live-attenuated VSVs has significantly improved the safety of the VSV therapy [2,3,4,7], anti-VSV drugs may further decrease the risk of the potential side effects caused by VSV replication after cancer treatment or vaccination. Such drugs may be necessary, if unanticipated dissemination of live-attenuated VSV itself or its pathogenic revertant occurs in cancer patients and, particularly, immunocompromised individuals.

VSV possesses a multifunctional RNA-dependent RNA polymerase (RdRp) L protein, which catalyzes all enzymatic reactions required for transcription and replication (reviewed in [16,17]). During transcription, the VSV L protein complexed with its cofactor P protein synthesizes the leader RNA (LeRNA, 47 nucleotides (nt)) and 5′-capped and 3′-polyadenylated mRNAs from the genome encapsidated with the N proteins (called N–RNA template) by a stop-start transcription mechanism [18,19,20,21]. A GDP polyribonucleotidyltransferase (PRNTase) domain in the L proteins of rhabdoviruses, such as VSV, Chandipura virus (CHPV), and RABV, carries out not only unconventional mRNA capping [22,23,24,25], but also transcriptional control [26,27,28,29]. Since the RdRp and PRNTase [26], but not methyltransferase [30,31], activities of the L protein are essential for VSV propagation in host cells, the RdRp and PRNTase domains are attractive targets for developing anti-rhabdoviral agents.

In this study, to screen a small-molecule library for anti-VSV compounds, we established a VSV cell killing assay that monitors VSV-induced cytopathic effects (CPEs) by using a cell viability assay with the Cell Counting Kit-8 (CCK-8) reagent [32]. Using the cell killing assay and following in vitro VSV transcription assay, we identified structurally related compounds (named vesiculopolins, VPIs) that inhibit VSV RNA synthesis as well as VSV-induced CPEs. We demonstrated that VPI A (the most effective compound) inhibits transcription initiation, but not mRNA capping, with the RNA-dependent RNA polymerase (RdRp) L protein of VSV. Furthermore, VPI A showed a weak inhibitory activity against transcription by the L protein of CHPV, which is closely related to VSV and associated with acute encephalitis in children with high mortality rate [33,34].

## 2. Materials and Methods

### 2.1. Chemicals

A small-molecule library composed of 50,000 structurally diverse compounds and selected compounds (see Table 1) were acquired from ChemBridge Corporation (San Diego, CA, USA) by the Small Molecule Drug Development Core (Case Western Reserve University). Ribavirin was purchased from Cayman Chemical (Ann Arbor, MI, USA). Nucleotides were obtained from Trilink Biotechnologies (San Diego, CA, USA). [α-^32^P]GTP and [α-^32^P]CTP were from PerkinElmer (Waltham, MA, USA).

### 2.2. Viruses

Recombinant VSV (wild-type, Indiana) and VSV expressing AcGFP (VSV-AcGFP) were generated from the pVSV-FL2 plasmid [35] and a modified pVSV-FL2 plasmid with an additional *AcGFP* gene unit (derived from pAcGFP-C1, Takara Bio USA, Mountain View, CA, USA) between the *N* and *P* genes, respectively, as described in [26,36]. Please note that the full-length VSV genome encoded by the pVSV-FL2 plasmid has been used as a backbone to develop oncolytic virus candidates [2,37,38,39] as well as vaccine candidates [7,40,41,42]. VSV and CHPV (653514 strain, VR-476, ATCC, Manassas, VA, USA) were propagated in BHK-21 cells (CCL-10, ATCC). Human parainfluenza virus 3 (HPIV-3, 47855 strain) was propagated in HeLa cells (CCL-2, ATCC). Recombinant RABV strain Nishigahara expressing luciferase (Ni-Luc) was generated as described previously [43].

### 2.3. VSV Cell Killing Assay in a 384-Well Format

BHK-21 cells (4 × 10^3^ cells, 20 µL Dulbecco’s Modified Eagle’s Medium (DMEM, Thermo Fisher Scientific, Waltham, MA, USA) supplemented with 7.5% heat-inactivated fetal bovine serum (FBS, Thermo Fisher Scientific)) were seeded in each well of transparent 384-well plates using a MultiFlo FX multi-mode reagent dispenser (BioTek, Winooski, VT, USA). Using a JANUS automated liquid handling workstation with a 384-pin tool (PerkinElmer), ~50 nL of each small-molecule compound (10 mM, dissolved in dimethyl sulfoxide (DMSO)) or DMSO was added to each well of the plates. Using the BioTek dispenser, 10 µL of a VSV dilution in DMEM (4 × 10^4^ plaque-forming units/mL) was added to each well to infect the cells at a multiplicity of infection (MOI) of 0.1. Control wells for mock-infection received 10 µL of DMEM culture medium. The plates were agitated using a microplate mixer. The final concentrations of the compounds and DMSO were ~17 µM and ~0.17%, respectively. The cells were incubated at 37 °C in a CO_2_ incubator. At 24 h post-infection, 10 µL of 2% CCK-8 (Dojindo Molecular Technologies, Rockville, MD, USA) diluted in DMEM was added to each well. After incubation of the cells for 1.5–2 h at 37 °C in the CO_2_ incubator, the absorbance at 450 nm (A_450_) was measured using an EnSpire multimode plate reader (PerkinElmer). Mean (µ) and standard deviation (δ) values for A_450_ of mock (m)- and VSV (v)-infected cell control wells (*n* = 32) were determined for 157 plates. The Z′ factor [44,45] for each plate was calculated using the formula: 1 − (3δ_m_ + 3δ_v_) / (µ_m_ − µ_v_). The percent inhibition of VSV-induced metabolic shut-down was calculated using the following formula: 100 × (A_450_ of each sample well − µ_v_) / (µ_m_ − µ_v_). After measuring the absorbance, the cells were fixed by adding 5 µL of 37% formaldehyde containing 40 µg/mL Hoechst 33342 dye (Sigma-Aldrich, St. Louis, MO, USA) [46]. VSV-induced nuclear condensation was analyzed using an Operetta high-content imaging system with a 20× objective (PerkinElmer) according to the manufacturer’s instructions for an apoptosis cell imaging assay (PerkinElmer). Image analyses and calculations were performed using Acapella and Harmony 4.1 software packages (PerkinElmer). Compounds displaying 30% or more inhibition of VSV-induced metabolic shut-down and nuclear condensation were selected as hits. Cells in hit wells were also observed under a microscope to confirm inhibition of VSV-induced cell rounding.

### 2.4. VSV Cell Killing Assay in a 96-Well Format

BHK-21 cells (2.5 × 10^4^ cells, 50 µL DMEM supplemented with 10% FBS) were seeded in each well of transparent 96-well plates. After the addition of 25 µL of each small molecule (0.78–400 µM, 0.8% DMSO) in DMEM or 0.8% DMSO in DMEM to each well of the plates, 25 µL of a virus dilution (VSV or CHPV) in DMEM (1 × 10^5^ plaque-forming units/mL) was added to each well to infect the cells at an MOI of 0.1. Control wells for mock-infection received 25 µL of DMEM culture medium. The final concentrations of each small molecule and DMSO were 0.2–100 µM and 0.2%, respectively. The cells were incubated at 37 °C in a CO_2_ incubator. At 24 h post-infection, 10 µL of CCK-8 was added to each well. After incubation of the cells for 1.5–2 h at 37 °C, the absorbance at A_450_ was measured using the Thermo Multiskan FC microplate photometer (Thermo Fisher Scientific). The percent inhibition of VSV-induced metabolic shut-down by various concentrations of small molecules was calculated as described in Section 2.3. Fifty percent inhibitory concentration (IC_50_) values were determined by fitting experimental data to the four-parameter logistic equation using Graphpad Prism software (version 7) (GraphPad Software, San Diego, CA, USA). The percent cell viability was calculated using the following formula: 100 × (A_450_ of each sample well − A_450_ of a well without cells (blank)) / (µ_m_ − blank). Similarly, HeLa cells (2.5 × 10^4^ cells) were infected with VSV at an MOI of 0.1 in the presence or absence of various concentrations of each small molecule in a 96-well plate, and cultured for 65 h. The cell viability was determined as described above, except that the cells were incubated with CCK8 for 40–60 min.

### 2.5. Other VSV Cell Infection Assays

BHK-21 cells (2.5 × 10^4^ cells) were infected with VSV at an MOI of 0.1 in the presence of various concentrations of each small molecule in a 96-well plate and incubated for 24 h as described in Section 2.4. The culture supernatants were subjected to a plaque assay to determine their virus titers [26]. Similarly, BHK-21 cells were infected with VSV-AcGFP and cultured for 24 h. After addition of Hoechst 33342 to culture medium to a final concentration of 5 µg/mL, cells were incubated for 30 min at 37 °C in a CO_2_ incubator, and then observed under a fluorescent microscope with a 20× objective (EVOS FLoid Cell Imaging Station, Thermo Fisher Scientific).

### 2.6. Preparation of Viral RNPs and Proteins

The VSV RNP, N–RNA template, and recombinant L and P proteins were prepared as described previously [22,23,47]. CHPV was propagated in BHK-21 cells and purified by equilibrium banding on a potassium tartrate-glycerol gradient essentially as described in [48]. CHPV RNP was purified from detergent-disrupted virus particles as described for VSV [22].

HeLa cells were infected with HPIV-3 at an MOI of 5, and cultured for 24 h. The infected cells were homogenized in the hypotonic buffer [49], and a post-nuclear supernatant was prepared from the cell homogenate and subjected to subcellular fractionation as described previously [49]. A microsome (P100) fraction (7.2 mg of protein) was incubated in 5 mL of a lysis buffer [20 mM Tris-HCl (pH 8.0), 100 mM NaCl, 1 mM MgCl_2_, 1 mM DTT, 2% Triton X-100) for 15 min at 4 °C, and centrifuged at 15,000 × *g* for 10 min. The resulting pellet containing HPIV-3 RNP was suspended in the isotonic buffer [49] to obtain a crude RNP fraction (2.8 mg protein/mL, 1 mL).

### 2.7. In Vitro Transcription and Capping Assays

In vitro transcription was performed with 0.2 µg protein of VSV or CHPV RNP in the presence of 0.1 mM [α-^32^P]GTP and 1 mM each of ATP, CTP, and UTP as detailed previously [47]. For HPIV-3, 3.5 µg protein of the crude RNP fraction was used for in vitro transcription. In vitro AC synthesis (first phosphodiester bond formation) was carried out with 2 mM ATP and 20 µM [α-^32^P]CTP as described in [28]. In vitro oligo-RNA capping was conducted with 0.25 µM [α-^32^P]GDP, 2.5 µM pppAACAG, and 60 ng of the recombinant L protein as described previously [50]. The above enzymatic reactions were performed in the presence of different concentrations of VPI A. All the reaction mixtures included 2% DMSO. ^32^P-Labeled RNA products were analyzed by electrophoresis in 5% (for mRNAs) or 20% (for LeRNA, ApC, and capped oligo-RNA) polyacrylamide gels containing 7 M urea (urea-PAGE) followed by autoradiography [47,50]. Statistical analyses were performed by one-way ANOVA using the Graphpad Prism software.

### 2.8. In Vitro Pulse-Chase Lerna Synthesis Assay

The recombinant L (0.15 µg) and P (0.05 µg) proteins were preincubated with N–RNA (0.4 µg protein) in the presence or absence of VPI A (final concentration: 1 or 10 µM) in 23 µL of the transcription buffer [47] without NTPs at 30 °C for 30 sec. After the addition of 2 µL of a mixture containing ATP, CTP (final concentration: 1 mM each), and [α-^32^P]GTP (final concentration: 1 µM, ~400 cpm/fmol), the reaction mixtures were incubated at 30 °C for 3 min (*pulse*). The reactions were terminated by the addition of the RNA extraction buffer [47]. RNA products including LeRNA with residues 1–18 (LeRNA_1–18_) were extracted, precipitated, and then analyzed by 20% urea-PAGE followed by autoradiography as described previously [47].

To analyze effects of VPI A on elongation of LeRNA_1–18_ to LeRNA (47 nt), ^32^P-labeled LeRNA_1–18_ was synthesized without any inhibitors as described above (*pulse*). After adding 5 µL of the transcription buffer containing GTP and UTP (final concentration: 1 mM each) with or without VPI A (final concentration: 1 or 10 µM) to the reaction mixtures, the reactions were continued for additional 1 min at 30 °C (*chase*) and then terminated. RNA products were analyzed as described above.

An oligo-DNA (5′-AATGGTTTGTTTGTCTTCGTTATAGTGAGTCGTATTA, template region underlined) was annealed to 5′-TAATACGACTCACTAT (class III T7 promoter sequence) and used as a template to synthesize a LeRNA_1–18_ marker with ATP, CTP, and [α-^32^P]GTP by T7 RNA polymerase as described before [47,51].

## 3. Results

### 3.1. Discovery of Vesiculopolins as VSV RdRp Inhibitors

**Figure 1:**
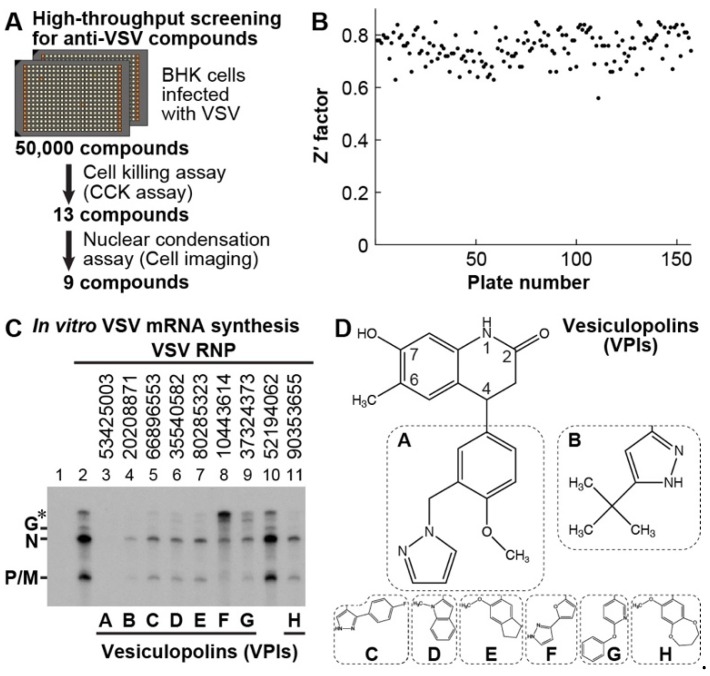
Screening for anti-VSV compounds. A flow diagram of screening for anti-VSV compounds is shown in (**A**). High-throughput screening of the ChemBridge library composed of 50,000 small molecules was performed for compounds that rescue cells from VSV-induced CPEs (metabolic shut-down and nuclear condensation). BHK-21 cells were mock-infected or infected with VSV at an MOI of 0.1 in the presence or absence of each compound (~17 µM) and cultured for 24 h. VSV-induced metabolic shut-down was measured as a decrease in cellular dehydrogenase activities by a colorimetric cell viability assay with the CCK-8 reagent (CCK assay). As described in Section 2.3, the Z′ factor for each plate was calculated (**B**). After fixing and staining the cells, cell nuclei were analyzed by an automated cell imaging system. Compounds displaying ≥30% inhibition of VSV-induced metabolic shut-down and nuclear condensation were selected as primary hits. In vitro mRNA synthesis was performed with VSV RNP in the presence or absence of the hit compounds (20 µM) (**C**). Deadenylated mRNAs were analyzed by 5% urea-PAGE followed by autoradiography. Lane 1 indicates no RNP. Eight-digit ChemBridge identification numbers for the hit compounds are shown above the lanes. The positions of the VSV mRNAs (N, P/M, and G) are indicated. Unknown transcripts of 2–3 kilo nt in length are marked by an asterisk. Eight structurally related compounds (named vesiculopolins, VPI A–H) were identified as VSV RdRp inhibitors. VPIs have a common structure [7-hydroxy-6-methyl-3,4-dihydroquinolin-2(1H)-one] with a variable group at position 4 (**D**). The chemical structure of VPI A is shown as a representative. For VPIs B–H, only substituent groups at position 4 are shown.

To find small molecules that rescue cultured BHK-21 cells (hamster kidney fibroblast cells) from VSV-induced CPEs (e.g., metabolic shut-down, nuclear condensation, cell rounding), we performed screening of the ChemBridge library composed of 50,000 drug-like molecules (final concentration: ~17 µM) by using the VSV cell killing assay in a 384-well format (Figure 1A). In this assay, VSV-induced metabolic shut-down was measured as a decrease in cellular dehydrogenase activities with the colorimetric CCK-8 reagent containing a water-soluble tetrazolium salt and electron mediator [32]. To evaluate the assay quality for every assay plate, we determined the Z′ factor [44,45] based on the absorbance of mock- and VSV-infected cell control wells (Figure 1B). The overall Z′ factor was 0.75 ± 0.06 (mean ± standard deviation, *n* = 157), indicating that excellent assay conditions were maintained throughout the screen. We identified 13 hit compounds with ≥30% inhibitory activities against VSV-induced metabolic shut-down. Although this screening system based on the rescue of cells from lytic VSV infection might eliminate compounds that are highly toxic to cells from primary hits, we further eliminated compounds that cannot repress VSV-induced apoptotic nuclear condensation by performing a cell imaging assay with the PerkinElmer Operetta. The result showed that 9 compounds (0.02 % hit rate) exhibited ≥30% inhibitory activities against VSV-induced nuclear condensation (Table 1).

Using our in vitro transcription system, we revealed that the top 2 compounds (Table 1; Figure 1C, lanes 3 and 4) strongly inhibit VSV mRNA synthesis at 20 µM, while other 6 compounds moderately or weakly repress mRNA synthesis (lanes 5–9 and 11). These 8 compounds were found to possess a common chemical structure, 7-hydroxy-6-methyl-3,4-dihydroquinolin-2(1H)-one, with an aromatic group at position 4 (Figure 1D). We named these compounds vesiculopolins (VPIs) A–H. Interestingly, VPI F exhibited a weak inhibitory effect on mRNA synthesis, but induced production of larger amounts of unknown transcripts with 2–3 kilo nt (Figure 1C, lane 8, marked by an asterisk) than in the absence of the compound (lane 2). There was only one hit compound, which was structurally unrelated to VPIs and had only a subtle or no effect on mRNA synthesis (Figure 1C, lane 10).

### 3.2. Antiviral Activities of Vesiculopolins A and B against Rhabdoviruses

**Figure 2:**
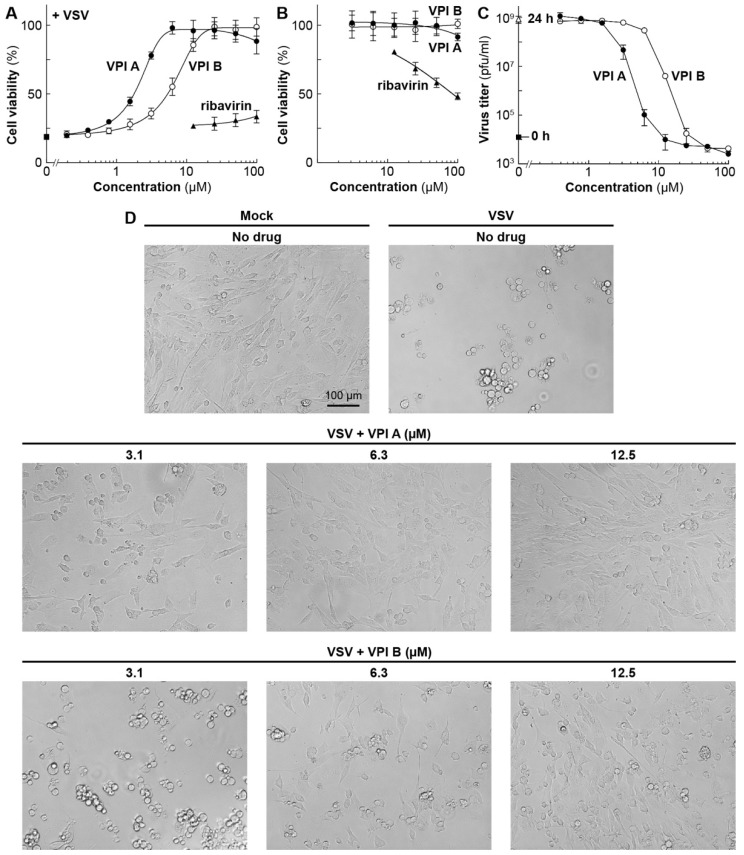
Anti-VSV activities of VPIs A and B in BHK-21 cells. BHK-21 cells in 96-well plates were mock-infected or infected with VSV at an MOI of 0.1 in the presence or absence of different concentrations of VPI A (closed circles), VPI B (open circles), or ribavirin (closed triangles), and cultured for 24 h. The viability of infected (**A**) or mock-infected (**B**) cells was assessed by the CCK assay. The viability of mock-infected cells (no drug) was set to 100%. Symbols and error bars represent the means and standard deviations, respectively, of three independent experiments, which were conducted in triplicate. In (**A**), the viability of infected cells (no drug) is shown by a closed square. VSV titers (plaque-forming units (pfu)/mL) of culture supernatants at 24 h post-infection were determined by a plaque assay (**C**). Symbols and error bars indicate the means and standard deviations, respectively, of three experiments. VSV titers of culture supernatants at 0 h and 24 h post-infection (no drug) are shown by a closed square and open triangle, respectively. The mock-infected or infected cells in the presence or absence of VPI A or B at various concentrations were observed under a microscope (**D**).

We further characterized VPIs A and B, because they showed more potent inhibitory activities against in vitro mRNA synthesis as well as VSV-induced CPEs among VPIs (Figure 1C and Table 1). VPIs A and B inhibited VSV-induced metabolic shut-down, with IC_50_ values of 2 and 6 µM, respectively (Figure 2A, Table 2), and have little or no cytotoxic effects on BHK-21 cells at a concentration of 100 µM (Figure 2B). In contrast, ribavirin, a broad-spectrum antiviral drug, exhibited significant cytotoxic effects (CC_50_: 86 µM) on BHK-21 cells (Figure 2B and Table 2), rather than anti-VSV effects (Figure 2A), at concentrations ranging from 25 to 100 µM.

**Figure 3:**
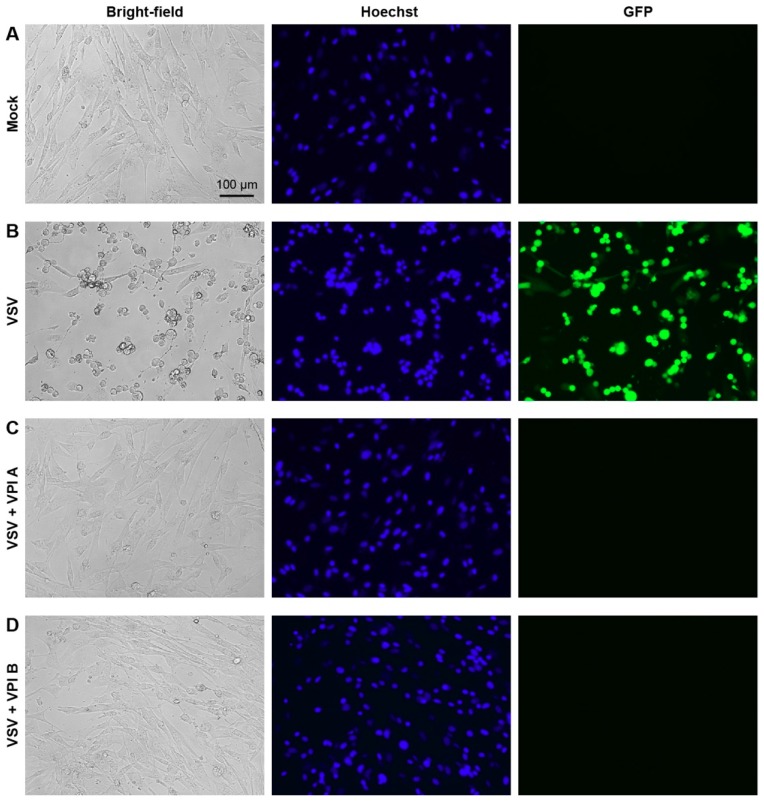
VPIs A and B inhibit VSV-induced CPEs and gene expression. BHK-21 cells were mock-infected (**A**) or infected with recombinant VSV expressing AcGFP at an MOI of 0.1 (**B**–**D**) in the presence or absence (**A**,**B**) of 10 µM VPI A (**C**) or B (**D**). At 24 h post-infection, cells were stained with Hoechst 33342, and observed under a fluorescent microscope.

VPIs A and B almost completely abolished VSV production from BHK-21 cells at concentrations of ≥12.5 and ≥25 µM, respectively (Figure 2C). Consistently, microscopic observations revealed that VPIs A and B repress VSV-induced cell rounding and detachment in a concentration-dependent manner, and nearly completely protected cells from these CPEs at concentrations of ≥12.5 and ≥25 µM, respectively (Figure 2D). We also confirmed that VPIs A and B abrogate nuclear condensation and AcGFP expression in BHK-21 cells infected with VSV with the *AcGFP* gene at 10 µM (Figure 3). Time-of-addition experiments revealed that VPI A inhibits VSV infection at an early post-entry step (Figure S1).

**Figure 4:**
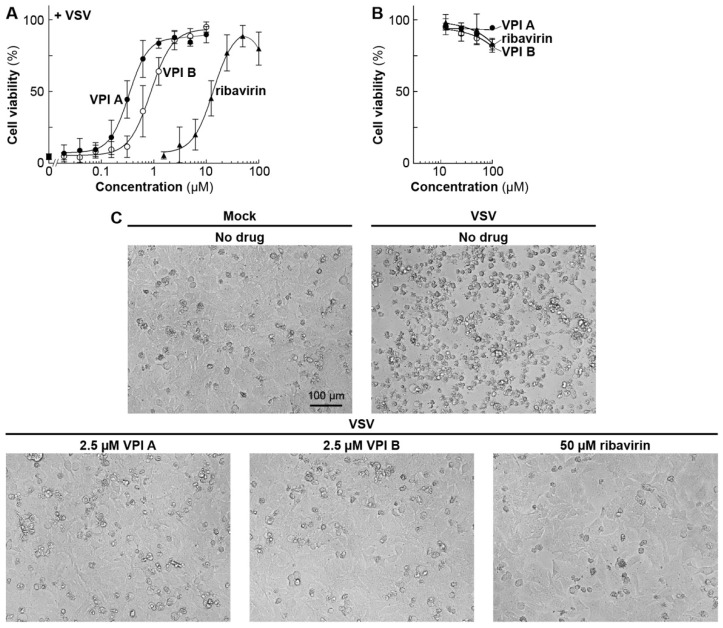
Anti-VSV activities of VPIs A and B in HeLa cells. HeLa cells in 96-well plates were mock-infected or infected with VSV at an MOI of 0.1 in the presence of different concentrations of VPI A (closed circles), VPI B (open circles), or ribavirin (closed triangles), and cultured for 65 h. The cell viability of infected (**A**) or mock-infected (**B**) cells was determined as described in Figure 2. The mock-infected or infected cells in the presence or absence of the indicated compounds were observed under a microscope (**C**).

VPIs A and B also inhibited VSV-induced metabolic shut-down in HeLa cells (human cervical cancer cells) with IC_50_ values of 0.4 and 1 µM, respectively (Figure 4A, Table 2) and cell rounding and detachment at 2.5 µM (Figure 4C), and were less toxic to the HeLa cells at 100 µM during the 65 h incubation period (Figure 4B). On the other hand, ribavirin protected HeLa cells from VSV infection at high concentrations (IC_50_: 15 µM) without significant cytotoxicity (Figure 4A–C), consistent with the reported cell-type-specific anti-VSV effects of ribavirin [52].

**Figure 5:**
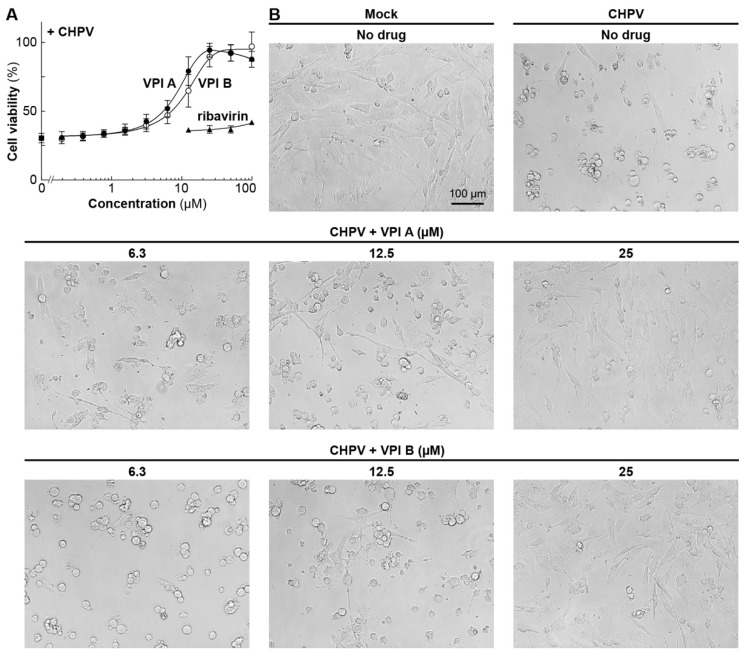
Anti-CHPV activities of VPIs A and B in BHK-21 cells. The viability of CHPV-infected BHK cells in the presence of different concentrations of VPI A, VPI B or ribavirin was determined by the CCK assay (**A**) as described in Figure 2. Indicated cells were observed under a microscope (**B**).

We analyzed antiviral activities of VPIs A and B against other rhabdoviruses. VPIs A and B inhibited CHPV-induced CPEs, such as metabolic shut-down (IC_50_: 8 and 11 µM, respectively) (Figure 5A, Table 2) and cell rounding and detachment (Figure 5B), although to lesser extents. VPI A also exhibited a weak inhibitory effect on RABV gene expression in infected mouse neuroblastoma NA cells (IC_50_: 26 µM) when using the cell infection assay with recombinant RABV expressing firefly luciferase [43] (Figure S2 and Table S1). In contrast, VPI B showed little or no inhibitory effect on RABV gene expression, even at 100 µM. Although these results cannot be directly compared, the antiviral effects of VPIs against RABV seem to be remarkably weaker than those against vesiculoviruses. On the other hand, neither of the compounds showed any antiviral effects against HPIV-3, a paramyxovirus, when using a cell killing assay for HeLa cells (Figure S3 and Table S2).

### 3.3. The Mode of Action of Vesiculopolin A in Inhibition of VSV RdRp

**Figure 6:**
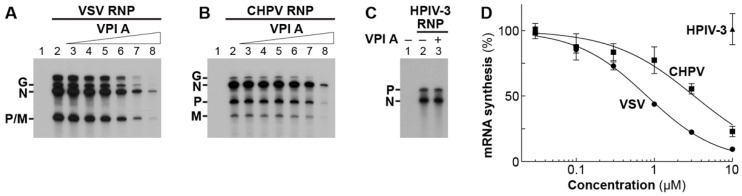
VPIs A and B inhibit mRNA synthesis of VSV and CHPV in vitro. In vitro mRNA synthesis was carried out with VSV (**A**), CHPV (**B**), or HPIV-3 (**C**) RNP in the presence or absence of VPI A (A and B, lanes 3–8, 0.03, 0.1, 0.3, 1, 3, 10 µM; C, lane 3, 10 µM). Deadenylated mRNAs were analyzed by 5% urea-PAGE followed by autoradiography. In (**D**), the graph shows relative activities for mRNA synthesis reactions, where radioactivities of transcripts synthesized without and with RNP (A–C, lanes 1 and 2, no drug) were set to 0 and 100%, respectively. Symbols and error bars represent the means and standard deviations, respectively, of three independent experiments.

**Figure 7:**
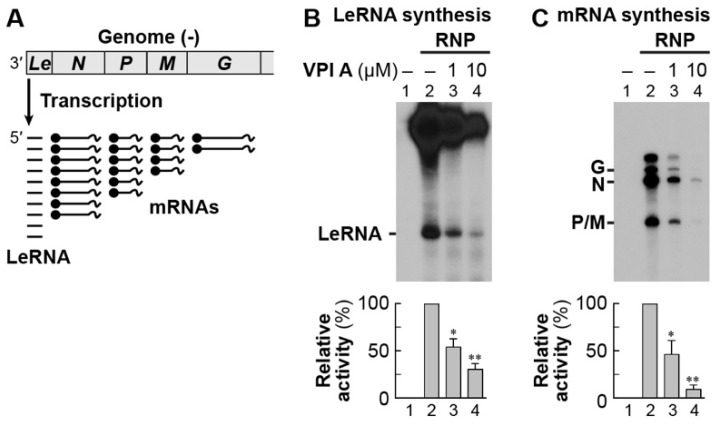
VPI A inhibits VSV LeRNA synthesis. As illustrated in (**A**), the VSV RdRp sequentially transcribes the genome in the N–RNA complex into the leader RNA (LeRNA) and 5′-capped and 3′-polyadenylated mRNAs. In vitro transcription was performed with VSV RNP in the presence or absence of 1 or 10 µM VPI A. LeRNA and deadenylated mRNAs were analyzed by 20% (**B**) or 5% (**C**) urea-PAGE followed by autoradiography. The graphs show relative RNA synthesis activities, where radioactivities of each product synthesized without and with RNP (B and C, lanes 1 and 2, no drug) were set to 0 and 100%, respectively. Statistical significance was determined by one-way ANOVA (*, *p* < 0.05; **, *p* <0.01; compared to control (column 2)).

To investigate the mode of action of VPIs, we used VPI A, the most potent anti-VSV compound among them. Consistent with the antiviral effects of VPI A, it inhibited in vitro mRNA synthesis of VSV (Figure 6A) and CHPV (Figure 6B), but not of HPIV-3 at 10 µM (Figure 6C). IC_50_ values against VSV and CHPV mRNA synthesis were calculated to be 0.81 ± 0.04 and 3.3 ± 0.4 µM (means ± standard errors, *n* = 3), respectively (Figure 6D). According to the single-entry, stop-start transcription model [19,20,21,53], the VSV RdRp complex initiates RNA synthesis at the 3′-end of the genome to sequentially synthesize LeRNA and mRNAs by following the gene order: *Le*-*N*-*P*-*M*-*G*-*L* (Figure 7A). We found that VPI A represses LeRNA synthesis (Figure 7B), as well as mRNA synthesis (Figure 7C), therefore suggesting that inhibition of LeRNA synthesis may cause the reduction in mRNA synthesis from the internal genes in an indirect manner.

**Figure 8:**
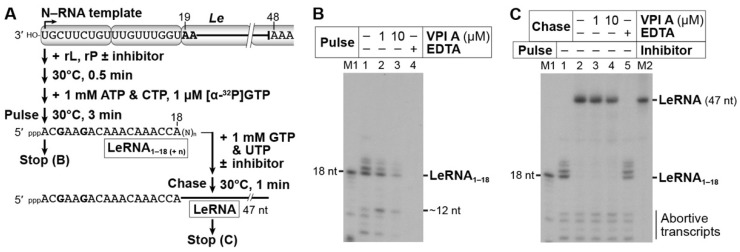
VPI A inhibits an early step of VSV LeRNA synthesis. The experimental design for pulse-chase transcription is schematically represented in (**A**). Partial RNA sequences of the 3′-terminal VSV genome and 5′-terminal LeRNA are shown. The recombinant VSV L and P proteins was premixed with the N–RNA template in the presence or absence of VPI A (1 or 10 µM) or EDTA (25 mM). After adding ATP, CTP, and [α-^32^P]GTP to the reactions, ^32^P-labeled LeRNA with residues 1–18 (LeRNA_1–18_) was synthesized for 3 min (*pulse*). The reactions were stopped (**B**) or proceeded to further RNA chain elongation with excess concentrations of GTP and UTP in the presence or absence of VPI A (1 or 10 µM) or EDTA (25 mM) (**C**, *chase*). Transcripts were analyzed by 20% urea-PAGE followed by autoradiography. M1 and M2 lanes indicate ^32^P-labeled LeRNA_1–18_ and LeRNA synthesized by T7 RNA polymerase and VSV RNP, respectively.

To further investigate the effects of VPI A on different stages of LeRNA synthesis, we established a pulse-chase LeRNA synthesis assay. In this assay, an RdRp complex was preassembled with the recombinant L and P proteins on the N–RNA template (Figure 8A). By incubating the complex with ATP, CTP, and [α-^32^P]GTP (*pulse*), the reconstituted RdRp complex was expected to initiate terminal de novo initiation for LeRNA synthesis and subsequently to elongate nascent LeRNA. Then, the RdRp was anticipated to pause at position 18 due to a lack of UTP, which serves as an incoming nucleotide complementary to the A residue at position 19 of the genome. If the RdRp formed a stable elongation complex, ^32^P-labeled LeRNA with residues 1–18 (LeRNA_1–18_) would be elongated to full-length LeRNA (47 nt) by the addition of excess concentrations of GTP and UTP (*chase*). As expected, during the pulse period, the reconstituted RdRp complex produced ^32^P-labeled LeRNA_1–18_, but also synthesized transcripts longer than expected (19–21 nt) (Figure 8B,C, lane 1). Since T7 RNA polymerase was able to terminate transcription at position 18 on the DNA template (see Section 2.8.) without UTP to produce LeRNA_1–18_ (Figure 8B, lane M1), ATP, CTP, and GTP used for transcription seemed to be UTP free. Therefore, these observations suggest two possibilities: the VSV RdRp incorporated UMP from a trace amount of UTP co-purified with the viral proteins or misincorporated AMP, CMP, or GMP into the 3′-end of LeRNA_1–18_. Nevertheless, the VSV RdRp elongated the transcripts of 18–21 nt, but not transcripts of 8–12 nt (abortive transcripts), to full-length LeRNA (47 nt) during the chase period in the presence of GTP and UTP (Figure 8C, lane 2), indicating that active elongation complexes were successfully formed during the pulse period. Using this system, we revealed that VPI A inhibits synthesis of LeRNA_1–18_ (Figure 8B, lanes 2 and 3), rather than its elongation to full-length LeRNA (Figure 8C, lanes 3 and 4), whereas the divalent metal ion chelator EDTA abolishes both the reactions (Figure 8B, lane 4; Figure 8C, lane 5). These results suggest that VPI A affects an early step(s) of LeRNA synthesis, such as transcription initiation and transition from initiation to processive elongation. Since a transcript of ~12 nt appeared to be accumulated in the presence of 1 µM VPI A during the pulse period (Figure 8, lane 2), VPI A was thought to affect an early phase of RNA chain elongation to some extent.

**Figure 9:**
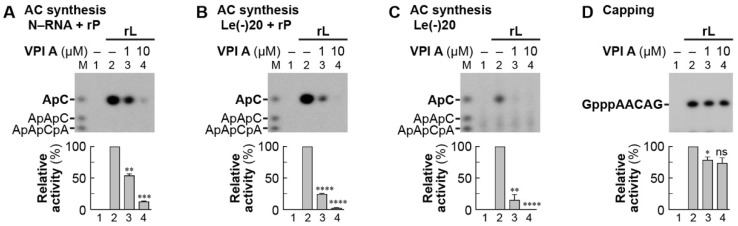
VPI A inhibits terminal de novo initiation of LeRNA synthesis with the VSV L protein. In vitro AC synthesis (first phosphodiester bond formation) by the recombinant VSV L protein was performed with (**A**,**B**) or without (**C**) the P protein in the presence or absence of VPI A (1 or 10 µM). The N–RNA (A) or Le(-)20 oligo RNA (**B**,**C**) was used as a template. Calf intestine alkaline phosphatase-resistant RNA products were analyzed by 20% urea-PAGE followed by autoradiography. Lane 1 indicates no L protein. M lane shows 5′-hydroxyl oligo-RNAs with the indicated sequences. In (**D**), in vitro oligo-RNA capping by the recombinant VSV L protein was carried out with [α-^32^P]GDP and pppAACAG in the presence or absence of VPI A (1 or 10 µM). The graphs show relative activities of AC synthesis (**A**–**C**) and oligo-RNA capping (**D**), where radioactivities of each product synthesized without and with the L protein (lanes 1 and 2, no drug) were set to 0 and 100%, respectively. Statistical significance was determined by one-way ANOVA (ns, not significant (*p* < 0.5); *, *p* < 0.05; **, *p* <0.01; ***, *p* < 0.001; ****, *p* < 0.0001; compared to control (column 2)).

Finally, to analyze the effects of VPI A on terminal de novo initiation for LeRNA synthesis, we performed the first phosphodiester bond formation (AC synthesis) using the N–RNA or a 20-nt oligo-RNA with the *Le* promoter sequence (Le(-)20) as a template [28]. VPI A was found to inhibit AC synthesis with the VSV L and P proteins from either template (Figure 9A,B, lanes 3 and 4), indicating that the N protein is not a target of VPI A. VPIs B–H also inhibited the first phosphodiester bond formation (Figure S4), consistent with their inhibitory activities against VSV mRNA synthesis (Figure 1 and Table 1). In addition, VPI A was found to inhibit de novo transcription initiation catalyzed by the CHPV RdRp, as well (Figure S5). Although the VSV L protein alone showed a ~75-fold lower AC synthesis activity than in the presence of the P protein [28], it synthesized a detectable amount of AC (Figure 9C, lane 2). Using this system, we showed that VPI A inhibits AC synthesis with the VSV L protein alone by 85 and 100% at concentrations of 1 and 10 µM, respectively (Figure 9C, lanes 3 and 4). In contrast, VPI A slightly reduced the capping activity of the PRNTase domain in the VSV L protein even at 10 µM (Figure 9D, lane 4). Taken together, these results suggest that VPI A blocks transcription initiation, rather than elongation, by binding to the RdRp domain or its vicinity in the VSV L protein.

## 4. Discussion

In this study, we identified structurally related anti-VSV compounds, named vesiculopolins (VPIs), as VSV RdRp inhibitors, in which VPI A with a 4-methoxy-3-(1H-pyrazol-1-ylmethyl)phenyl group at position 4 is the most potent compound (Table 1, Figure 1, Figure 2, Figure 3 and Figure 4). The aromatic groups at position 4 of VPIs largely affect their inhibitory activities against in vitro RNA synthesis as well as VSV-induced CPEs. It can be speculated that the common structure (7-hydroxy-6-methyl-3,4-dihydroquinolin-2(1H)-one) of VPIs is critical for binding to the L protein in the vicinity of its RdRp active site, whereas the distinct aromatic groups interfere with the functions of the RdRp domain differently possibly via steric hindrance. One unique observation was that VPI F with a 5-(1H-pyrazol-3-yl)-2-furyl group exhibits a weak inhibitory activity against terminal de novo initiation with the VSV RdRp (Supplementary Data, Figure S4), but induces the production of the unknown transcripts of 2–3 kilo nt in length (Figure 1C). Identification of the unknown transcripts will help understand the effects of VPI F on transcription. If these transcripts are generated by read-through of transcriptional stop-start signals on the genome, it would be interesting to elucidate the mechanism of the stop-start transcription using VPI F as a molecular probe. Structure-activity relationship studies are definitely necessary to define the unique inhibitory modes of VPIs, as well as to design more potent anti-VSV drugs using VPIs as lead compounds.

VPI A showed a weak anti-CHPV activity (Figure 5, Table 2) and, to a significantly lesser extent, anti-RABV activity when compared with its anti-VSV activity (Supplementary Data, Figure S2 and Table S1). The VSV L protein shares 60% and 36% amino acid sequence identity with the CHPV and RABV L proteins, respectively, suggesting that amino acid sequence differences within VPI A-binding sites among the L proteins may affect its affinities for the respective proteins. Although VPI A and other related compounds may have the potential to serve as lead compounds to develop antiviral drugs against CHPV and RABV, these compounds should be differently modified to fit the respective L proteins.

We demonstrated that VPI A inhibits terminal de novo initiation, rather than elongation, for VSV LeRNA synthesis (Figure 8 and Figure 9). Therefore, once the VSV L protein forms an elongation complex, VPI A is no longer able to block RNA chain elongation. Similarly, benzimidazole-based non-nucleoside hepatitis C virus RdRp inhibitors, which interact with the RdRp thumb subdomain, allosterically inhibit de novo initiation, but not processive elongation [54,55,56]. We have recently discovered that terminal de novo initiation at the 3′-end of the VSV genome requires a highly conserved tryptophan residue on the priming-capping loop [28], which is extended from the PRNTase domain into the RdRp active site cavity of the L protein. VPI A may interfere with an initiation-specific event, such as the initiation complex assembly on the 3′-terminal *Le* promoter of the genomic RNA and following first phosphodiester bond formation mediated by the priming-capping loop. LeRNA synthesis is a prerequisite for mRNA synthesis from the internal genes by stop-start transcription in vitro [53]. Thus, it can be assumed that inhibition of mRNA synthesis by VPI A is a secondary effect of inhibition of LeRNA synthesis (Figure 6 and Figure 7), although we cannot rule out a direct effect of VPI A on internal de novo initiation for mRNA synthesis. In infected cells, VPI A may also abolish genome replication in the step of terminal de novo initiation. Further studies are currently underway to elucidate a type of RdRp inhibition by VPI A and to map its binding site in the VSV L protein.

Oncolytic virotherapy is an emerging approach to kill cancer cells with replication competent viruses. T-VEC, a genetically modified herpes simplex virus (HSV), has recently been approved by the U.S. Food and Drug Administration for the treatment of advanced melanoma (reviewed in [57]). One of the advantages of HSV based oncolytic viruses is the availability of anti-HSV drugs, such as acyclovir, that can render them harmless to patients when unanticipated dissemination of infection occurs. Similarly, anti-VSV drugs would enable control of VSV infection in patients, and thereby add further safety to the VSV therapy. Recently, Maraba vesiculovirus (MARAV), a cousin of VSV, has been proven to be a potent oncolytic virus in various preclinical cancer models (reviewed in [58]). Since the VSV and MARAV L proteins share 78% amino acid sequence identity, it would be interesting to investigate antiviral activities of VPIs against MARAV. These compounds are expected to serve as antidotes against these potent oncolytic vesiculoviruses. Further studies are needed to evaluate the effectiveness and safety of VPIs as anti-vesiculoviral agents in vivo using animal models.

In conclusion, we identified a new class of anti-VSV compounds by performing screening of small molecules. VPI A was found to inhibit the RNA synthesis activity of the L protein in the step of terminal de novo initiation. These compounds may be useful for developing drugs against vesiculoviruses, such as VSV, MARAV, and CHPV, and, by extension, other pathogenic human rhabdoviruses, such as RABV and Bas-Congo virus (associated with acute hemorrhagic fever [59]) as lead compounds. These compounds may also serve as molecular probes to elucidate the mechanisms of rhabdoviral RNA synthesis.

## Figures and Tables

**Table 1:** Results of primary screening for anti-VSV compounds.

			% Inhibition		
	ChemBridge ID #	VPI	Metabolic Shut-down ^1^ (CCK Assay)	Nuclear Condensation ^1^ (Cell Imaging)	mRNA Synthesis ^2^ (*In vitro* Transcription)
1	53425003	A	105	98	99
2	20208871	B	102	92	89
3	66896553	C	58	63	72
4	35540582	D	55	57	72
5	80285323	E	50	94	72
6	10443614	F	48	69	34
7	37324373	G	46	54	68
8	52194062		44	51	10
9	69805228		39	0	n.d.
10	43534388		35	10	n.d.
11	90353655	H	34	53	73
12	81699442		31	0	n.d.
13	66349773		30	9	n.d.

^1^ Compound concentration: ~17 µM; ^2^ Compound concentration: 20 µM; n.d.: not determined.

**Table 2:** Anti-vesiculoviral activities of VPI A, VPI B, and ribavirin.

Compound	BHK-21 Cells					HeLa Cells		
	Cytotoxicity	Anti-VSV Activity		Anti-CHPV Activity		Cytotoxicity	Anti-VSV Activity	
	CC_50_^1^	IC_50_^1^	SI ^2^	IC_50_^1^	SI ^2^	CC_50_^1^	IC_50_^1^	SI ^2^
VPI A	>100	2.0 ± 0.1	>50	7.9 ± 0.4	>13	>100	0.42 ± 0.02	>240
VPI B	>100	6.2 ± 0.2	>16	11 ± 1	>9	>100	1.1 ± 0.1	>91
Ribavirin	86 ± 0.5	>100	<1	>100	<1	>100	15 ± 1	>7

^1^ Values (µM) represent means ± standard errors of the means (*n* = 9). ^2^ Selectivity index (SI) was calculated by dividing the CC_50_ values by the IC_50_ values.

## Supplementary Materials

The following are available online at https://www.mdpi.com/1999-4915/11/9/856/s1, Figure S1: VPI A inhibits VSV infection at an early post-entry step. Figure S2: Anti-RABV activities of VPIs A and B in NA cells., Figure S3: Anti-HPIV-3 activities of VPI A, VPI B, and ribavirin in HeLa cells., Figure S4. VPIs inhibit terminal de novo initiation of VSV RNA synthesis. Figure S5. VPI A inhibits terminal de novo initiation of CHPV RNA synthesis. Table S1: Anti-RABV activities of VPIs A and B, Table S2: Anti-HPIV-3 activities of VPI A, VPI B, and ribavirin.
