# Supplementary material for: Vesiculopolins, a New Class of Anti-Vesiculoviral Compounds, Inhibit Transcription Initiation of Vesiculoviruses
Source: Viruses. 2019 Sep 14;11(9):856. doi: 10.3390/v11090856 (PMC6783830; doi:10.3390/v11090856)
Supplement: Supplementary file 1 [file viruses-11-00856-s001.pdf]

Supplementary data

Vesiculopolins, a new class of anti-vesiculoviral compounds, inhibit transcription initiation of vesiculoviruses

Minako Ogino [1], Yuriy Fedorov [2], Drew J. Adams [3], Kazuma Okada [4,†], Naoto Ito [4,5], Makoto Sugiyama [4], and Tomoaki Ogino [1,6,*]

[1] Department of Molecular Biology and Microbiology, Case Western Reserve University School of Medicine, Cleveland, OH 44106, USA
[2] Small Molecule Drug Development Core, Case Western Reserve University School of Medicine, Cleveland, OH 44106, USA
[3] Department of Genetics and Genome Sciences, Case Western Reserve University School of Medicine, Cleveland, OH 44106, USA
[4] Laboratory of Zoonotic Diseases, Faculty of Applied Biological Sciences, Gifu University, 1-1 Yanagido, Gifu 501-1193, Japan
[5] Gifu Center for Highly Advanced Integration of Nanosciences and Life Sciences (G-CHAIN), Gifu University, 1-1 Yanagido, Gifu 501-1193, Japan
[6] Department of Inflammation and Immunity, Lerner Research Institute, Cleveland Clinic, Cleveland, OH 44195, USA

† Present address: Division of Microbiology, Osaka Institute of Public Health, Japan
* Correspondence: tomoaki.ogino@case.edu; Tel.: +1-216-368-2070

5. Supplementary materials and methods

5.1. Time-of-addition of VPI A to VSV-infected cells

BHK-21 cells (5 × 10^4 cells) were infected with VSV-AcGFP at an MOI of 10 in the presence or absence of 10 μM VPI A in 20 μl of DMEM containing 0.2% DMSO in a 96-well plate for 1 h at 37°C in a CO_2 incubator. During this period, the plate was rocked every 10 min. After washing the cells with 100 μl of DMEM three times, the cells were cultured in 100 μl of DMEM containing 5% FBS and 0.2% DMSO for 12 h in the presence or absence of 10 μM VPI A (Supplementary Figure S1). To add VPI A to the culture at the indicated time points, the culture medium (DMEM/5% FBS/0.2% DMSO) was replaced with that containing VPI A, and then the cells were continuously cultured. When the culture medium containing VPI A was removed at 3 h post-infection, cells were washed with 100 μl of DMEM three times, and then cultured in 100 μl of the culture medium for additional 9 h. The cells were observed under the fluorescent microscope as described in section 2.5 (see the main text).

5.2. RABV cell infection assay

VPIs A and B were evaluated for their anti-RABV activities using the cell infection assay with recombinant RABV (Nishigahara strain) expressing firefly luciferase (Ni-Luc) [43]. Briefly, mouse neuroblastoma NA cells (3 × 10^5 cells) were infected with Ni-Luc at an MOI of 0.1 and cultured in the presence or absence of VPI A or B for 48 h. Luciferase activities of lysates of the cells were measured by using Luciferase Assay System (Promega). Cytotoxicity of the compounds to NA cells were examined by Cell Titer Glo 2.0 Assay (Promega).

5.3. HPIV-3 cell killing assay

HeLa cells (2.5 × 10^4 cells) were infected with HPIV-3 at an MOI of 0.1 in the presence or absence of various concentrations of each small molecule in a 96-well plate and incubated for 72 h. The cell viability was determined as described in section 2.4 (see the main text).

5.4. Expression and purification of recombinant CHPV P and L proteins

The CHPV *P* and *L* open reading frames were amplified from the CHPV genome (653514 strain, ATCC VR-476, GenBank accession no.: MN147865) by RT-PCR, and cloned into a modified pFastBac1 (Thermo Fisher Scientific) plasmid for expression of a carboxyl-terminal octahistidine-tagged protein [24]. Recombinant baculoviruses expressing the CHPV L and P proteins were generated according to the protocol for the Bac-To-Bac baculovirus expression system (Thermo Fisher Scientific), and the respective proteins were expressed in Sf21 insect cells and purified using nickel-nitrilotriacetic acid-agarose resin (Qiagen) as described previously [24, 47]. Purified recombinant proteins were analyzed together with purified CHPV particles by electrophoresis in a 10% SDS-polyacrylamide gel (SDS-

PAGE) with a low degree of crosslinking (acrylamide-bisacrylamide ratio of 30:0.4) as described in [47].

5.5. In vitro AC synthesis with the CHPV L and P proteins

In vitro AC synthesis was performed with the recombinant CHPV L (0.15 μg) and P (10 ng) proteins using an oligo-RNA template [CHPV Le(-)20: 5′-AUU GGU UUG UUU UUC UUC GU-3′] as described for VSV [28].

6. Supplementary results

6.1. VPI A inhibits VSV infection at an early post-entry step

To speculate which step of VSV infection is inhibited by VPI A, we performed time-of-addition experiments (Figure S1). To analyze the effects of VPI A on single-round VSV infection, BHK-21 cells were infected with VSV expressing AcGFP at a high MOI (= 10) for 1 h, and washed to remove residual VSV particles in culture supernatants. Then, the infected cells were cultured in a fresh medium for 12 h until moderate CPEs and AcGFP expression were observed in the infected cells. While VPI A did not inhibit VSV infection when present during the VSV adsorption/entry step (Figure S1C), it inhibited VSV infection when added immediately after VSV entry (at 0 h post-infection) (Figure S1D). However, VPI A showed weak and almost no inhibitory effects on VSV infection when added at 3 h (Figure S1E) and 6 h (Figure S1F) post-infection, respectively. In contrast, the presence of VPI A during the first 3 h post-infection after the entry step significantly inhibited or delayed VSV infection even if the compound was washed out at 3 h post-infection (Figure S1G). These results suggest that VPI A inhibits VSV infection at an early post-entry step, such as transcription, consistent with its inhibitory effect on *in vitro* synthesis of VSV mRNAs as well as LeRNA (see Figures 6–9 in the main manuscript).

6.2. Anti-viral activities of VPIs A and B against RABV and HPIV-3

To analyze antiviral specificities of VPIs A and B against NNS RNA viruses, we examined their effects on RABV (*Lyssavirus*, *Rhabdoviridae*) and HPIV-3 (*Respirovirus*, *Paramyxoviridae*) infection. Although VPI A showed a very weak inhibitory activity against RABV gene expression in mouse neuroblastoma NA cells (IC_{50}: 26 μM), VPI B did not show any anti-RABV activity at 100 μM (Figure S2 and Table S1). On the other hand, both VPIs A and B were not able to inhibit HPIV-3-induced CPEs on HeLa cells at 50–100 μM (Figure S3 and Table S2), whereas ribavirin inhibited the CPEs although at higher concentrations (IC_{50} : 21 μM). These results indicate that VPIs A and B specifically inhibit infection with vesiculoviruses (see Figures 2–5 in the main manuscript).

6.3. VPIs inhibit terminal de novo initiation of VSV transcription

To investigate the effects of VPIs on terminal *de novo* initiation of VSV transcription, we performed AC synthesis in the presence of each VPI. Consistent with their inhibitory activities against mRNA synthesis (see Figure 1 in the main manuscript), all the VPIs (Figure S4, lanes 3–9, 11), but not the unrelated hit compound (lane 10), repressed AC synthesis. VPI A showed the strongest inhibitory activity (lane 3), whereas VPI F exhibited the weakest activity (lane 8) among VPIs.

6.4. VPI A inhibits terminal de novo initiation of CHPV transcription

Finally, we established an *in vitro* terminal *de novo* initiation assay for recombinant forms of the CHPV L and P proteins to examine the effect of VPI A on the first phosphodiester bond formation by the CHPV RdRp. The recombinant CHPV P and L proteins were expressed in insect cells and affinity-purified (Figure S5A, lanes 3 and 4, respectively). As reported for VSV [28], the CHPV RdRp reconstituted with these recombinant proteins synthesized an AC dinucleotide from an oligo-RNA template with the CHPV *Le* promoter sequence (Figure S5B, lane 2). VPI A inhibited CHPV AC synthesis by 77% and 97% at concentrations of 1 (lane 3) and 10 (lane 4) μM, respectively, indicating that it inhibits the first phosphodiester bond formation by the CHPV RdRp as well.

References (see the main manuscript)

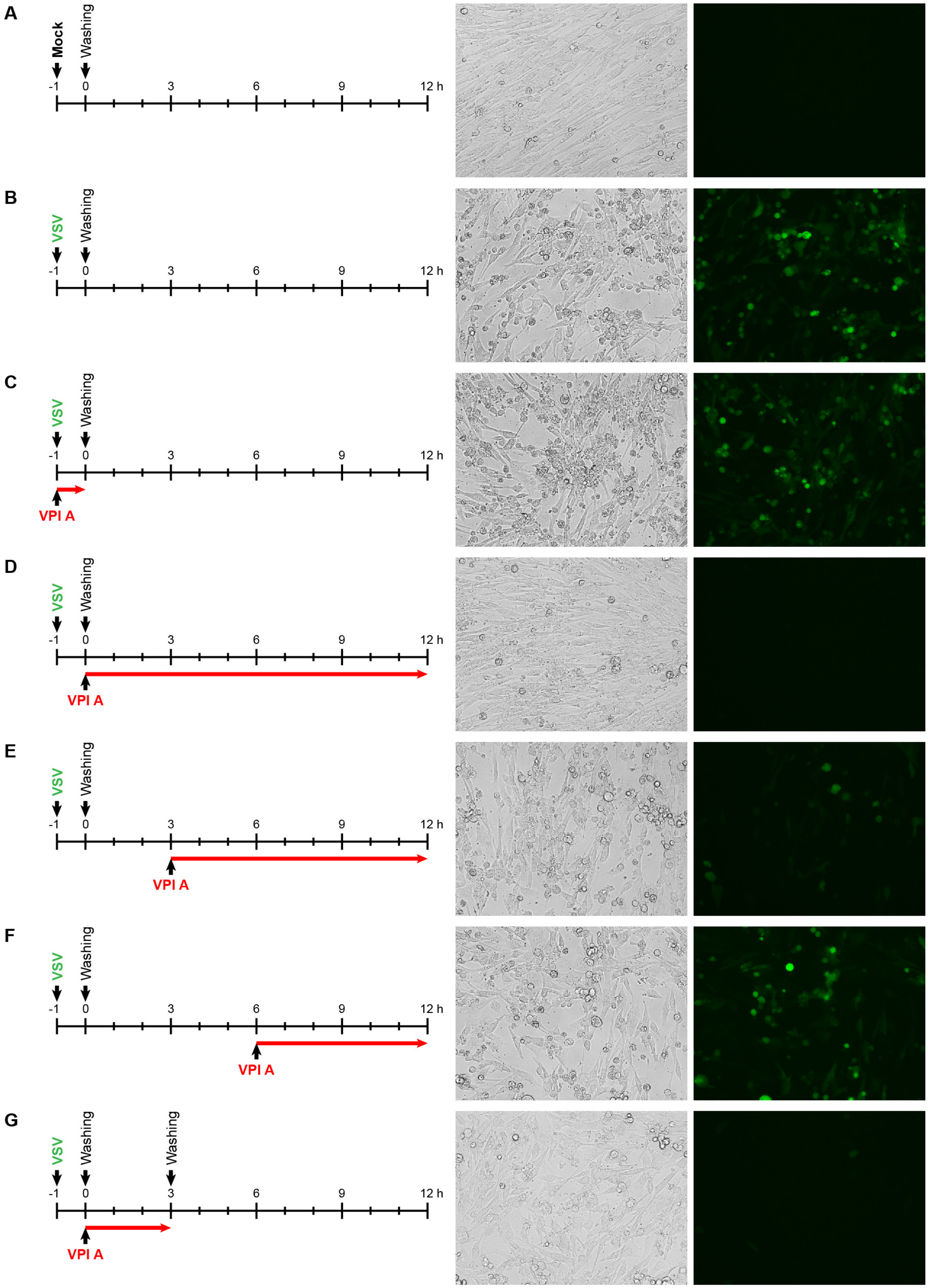

Figure S1. VPI A inhibits VSV infection at an early post-entry step. BHK-21 cells were mock-infected (**A**) or infected with recombinant VSV expressing AcGFP at an MOI of 10 in the presence (**C**) or absence (**B**, **D**–**G**) of 10 μM VPI A for 1 h at 37°C. After washing the cells, the cells were cultured in a fresh medium for 12 h in the presence or absence of 10 μM VPI A during the indicated time periods (left schematics). In (**G**), the infected cells were incubated with VPI A for the indicated period, washed, and then cultured in a fresh medium. At 12 h post-infection, the cells were observed under a fluorescent microscope. The middle and right panels show bright-field and green fluorescence images, respectively.

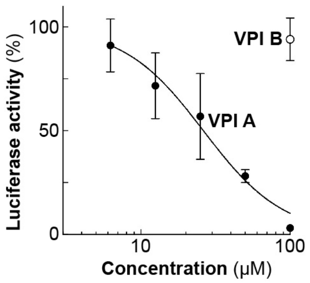

Figure S2. Anti-RABV activities of VPIs A and B in NA cells. NA cells were infected with RABV expressing luciferase at an MOI of 0.1 in the presence of different concentrations of VPI A (closed circles) or 100 μM VPI B (open circle), and cultured for 48 h. Luciferase activities of cell lysates were measured. The luciferase activity of the lysates of the cells infected with RABV in the absence of the compound was set to 100%. Symbols and error bars represent the means and standard deviations, respectively (n = 3).

Table S1

Anti-RABV activities of VPIs A and B

Compound	Cytotoxicity (NA)	Anti-RABV activity (NA)	
	CC_{50} [1]	IC_{50} [1]	SI [2]
VPI A	>100	26 ± 3	>4
VPI B	>100	>100	n.d.

[1] Values (μM) represent means ± standard errors of the means (n = 3).
[2] SI: CC_{50}/IC_{50}; n.d.: not determined.

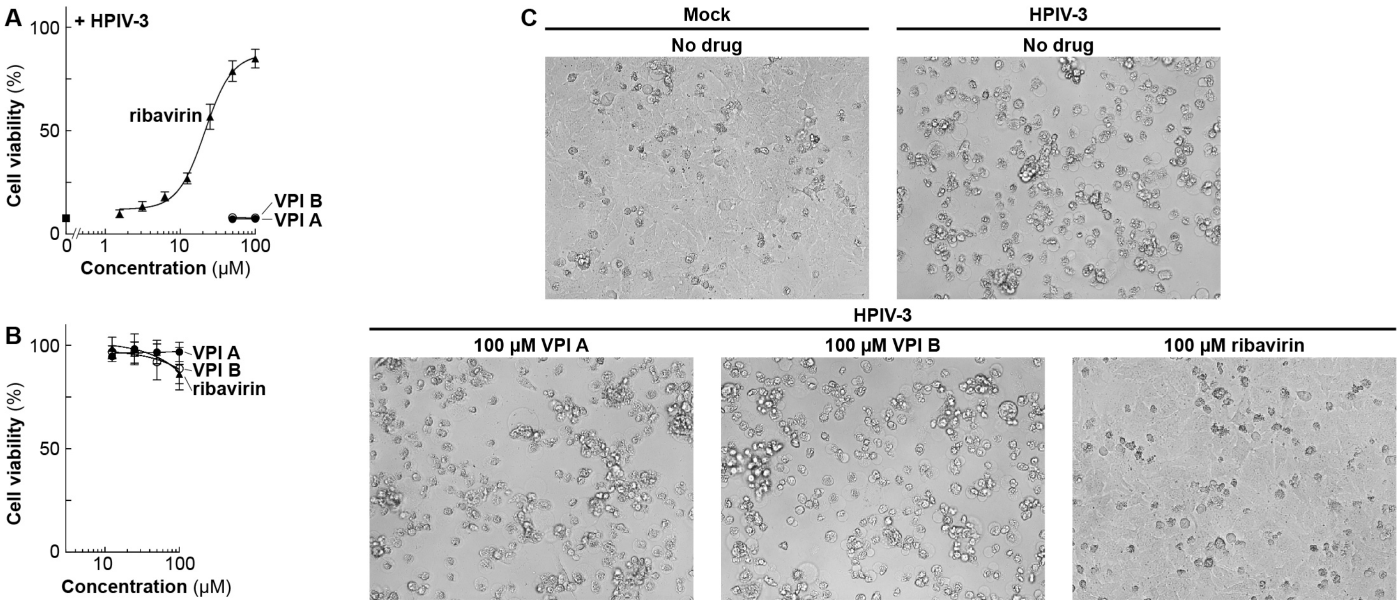

Figure S3. Anti-HPIV-3 activities of VPI A, VPI B, and ribavirin in HeLa cells. HeLa cells were mock-infected or infected with HPIV-3 at an MOI of 0.1 in the presence of different concentrations of VPI A (closed circles), VPI B (open circles), or ribavirin (closed triangles), and cultured for 72 h. The cell viability of infected (**A**) or mock-infected (**B**) cells was determined as described in Figure 2 (the main manuscript). The mock-infected or infected cells in the presence or absence of the indicated compounds were observed under a microscope (**C**).

Table S2

Anti-HPIV-3 activities of VPI A, VPI B, and ribavirin

Compound	Cytotoxicity (HeLa)	Anti-HPIV-3 activity (HeLa)	
	CC_{50} [1]	IC_{50} [1]	SI [2]
VPI A	>100	>100	n.d.
VPI B	>100	>100	n.d.
Ribavirin	>100	21 ± 1	>5

[1] Values (μM) represent means ± standard errors of the means (n = 9).
[2] SI: CC_{50}/IC_{50}; n.d.: not determined.

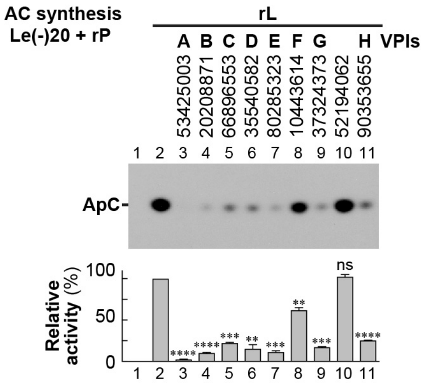

Figure S4. VPIs inhibit terminal *de novo* initiation of VSV RNA synthesis. *In vitro* AC synthesis was performed with the recombinant VSV L (rL) and P (rP) proteins and the Le(-)20 oligo-RNA template in the presence or absence of 10 μM VPIs A–H or the unrelated hit compound (see Figure 1 and Table 1 in the main manuscript) as described in Figure 9B (the main manuscript). Lane 1 indicates no L protein. The graph shows relative activities of AC synthesis, where radioactivities of the product synthesized without and with the L protein (lanes 1 and 2, no drug) were set to 0 and 100%, respectively. Statistical significance was determined by one-way ANOVA [ns, not significant ($P < 0.5$); **, $P < 0.01$; ***, $P < 0.001$; ****, $P < 0.0001$; compared to control (column 2)].

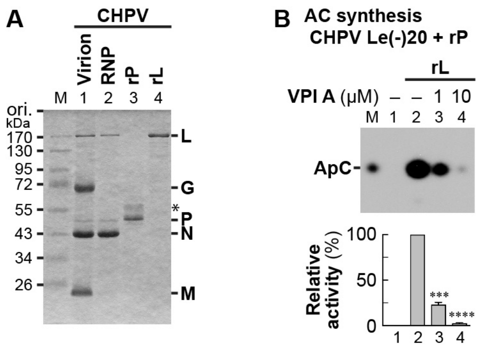

Figure S5. VPI A inhibits terminal *de novo* initiation of CHPV RNA synthesis. (**A**) CHPV virions (5 μg protein), RNP (3 μg protein), and recombinant P (rP, 1 μg) and L (rL, 1 μg) proteins were analyzed by 10% SDS-PAGE followed by Coomassie Brilliant Blue staining. The positions of marker proteins (M lane) and viral proteins are shown on the left and right, respectively. An asterisk indicates the position of a protein(s) co-purified with CHPV rP. (**B**) *In vitro* AC synthesis was performed with CHPV rL, rP, and the CHPV Le(-)20 oligo-RNA template in the presence or absence of the indicated concentrations of VPI A as described in Figure 9B (the main manuscript). M lane shows the 5′-hydroxyl AC marker. Relative activities of AC synthesis are shown in the graph as described in Figure S4.
